# A Severe Case of Puerperal Eclampsia at the Eighth Month of Pregnancy, with Recovery

**Published:** 1880-06

**Authors:** E. Day

**Affiliations:** Grand Tower, Illinois


### Clinical Reports.

---

### Notes From Private Practice.

### Article IV.

*A Severe Case of Puerperal Eclampsia at the Eighth Month of Pregnancy, with Recovery.*

I was called professionally, at one o'clock, on the morning of the 20th of January last, to visit Mrs. H.

On my arrival, I found her in a perfectly unconscious condition, having severe convulsions every few minutes, accompanied with frightful facial contortions; frothy mucus mixed with blood issued from the mouth; the pupils were dilated; the skin cool; pulse one hundred and sixty and feeble; and respiration stertorous and frequent. Her temperature was not noted. She was unable to swallow. From Mrs. C., at whose house Mrs. H. was residing temporarily, I obtained the following history of the case:

The patient is eighteen years of age; married, and within a month of expected confinement, primipara; has enjoyed good health to present illness; was taken sick during the preceding afternoon, and vomited several times; when, toward evening, slight labor pains ensued but irregularly. This continued to be the condition of Mrs. H. until about twelve o'clock, midnight, when she was seized with convulsions.

On examination per vaginam, I found a vertex presentation; os uteri dilated about two and a half centimeters in extent and patulous; and the pelvis of normal amplitude. Digital dilatation of the os was readily effected, and without much delay the membranes were ruptured and liquor amnii discharged. It now became apparent that the convulsions had entirely suspended uterine action, and, of course, no further advancement toward delivery could be expected under the circumstances, from the natural efforts of the patient. The extreme gravity of the case brooked no delay, and immediate delivery, became a *sine quâ non.*

Not having come prepared to encounter just such a case, some little time was consumed in sending for obstetrical instruments. Without any unnecessary delay, however, the forceps were applied, and the patient was readily delivered, at two o'clock a. m., of a dead female child, weighing eight pounds. The child had been dead, apparently, several hours.

The convulsions ceased, immediately after delivery, and did not return; respiration, gradually, became regular and free, and, indeed, all bad symptoms were at once ameliorated. After waiting an hour at the bedside, I administered an anodyne to control restlessness which began to manifest itself, and left the patient for the night. Her restoration to consciousness did not occur until seven o'clock in the morning, an interregnum of nearly eight hours. She made a speedy recovery, without the development of any untoward symptoms. The urine was not tested, but was no doubt highly albuminous, as is almost universally the case in this disease.

The notable points in this case, worthy of observation, are the extreme severity of the disease, the profound coma almost extinguishing life from the first, the sudden cessation of the convulsions after delivery, and the rapid recovery of the patient.

The usual treatment, restored to in puerperal eclampsia for the purpose of subduing the convulsions, viz., venesection, choloroform inhalation, chloral hydrate, etc., was not employed; and, I opine, would not have proved of much avail, as the vital powers were too much exhausted; besides, this would have consumed valuable time.

The conclusions of that eminent British obstetrician, Dr. Robert Barnes, in my opinion, establish the correct rule of procedure, in these cases, viz., That after the sixth month of pregnancy, the first, and indeed the most important measure, is to relieve the uterus of its burden, and this too at the earliest possible moment.

I am confident in considering the critical condition of the patient, when first seen, that nothing but delivery could have arrested the convulsions, and have prevented a fatal termination of the case. E. DAY, M.D.

GRAND TOWER, ILLINOIS.

---

TREATMENT OF PROLAPSUS OF THE RECTUM BY HYPODERMIC INJECTIONS OF ERGOTINE.—M. Vidal. (*Bulletin de l'Acad. de Méd.*, Feb. 3, 1880, P. 110.)

According to the author, prolapsus of the rectum may be readily cured, and in a comparatively short time, by hypodermic injections of ergotine. By this new method Vidal has succeeded in curing three adult cases, of which he gives the details. He used (gr. xv) one gram of Bonjean's extract of ergot or ergotine in (ʒ jss) five grams of cherry laurel water. Each injection consisted of 15 to 20 drops (exceptionally 25), which is equivalent to 20 to 25 centigrams of ergotine; or, in other words, to the extract of a gram and a half to three grams of ergot. None of the injections were followed by inflammation or abscess. Bonjean's ergotine causes a rather sharp, burning pain; the solution of iron is much better tolerated. In the future the author will give preference to the latter.

A CURIOUS ACOUSTIC ILLUSION. (*Telegraphic Journal*, London, September 15, 1879.) This illusion, pointed out by M. Plumaudon, of the Puy de Dôme Observatory, has also been observed by others who have made use of a pair of telephones in receiving messages or in experimental research. With a single telephone held, say, to the right ear, the transmitted voice seems to come from a distance to the right; while with a telephone held to the left ear it seems to arrive from the left of the listener. With a telephone to each ear, if one ear be less sensitive than the other, or if the telephone be held farther from that ear, the voice apparently shifts to the side of the other ear; and if both ears hear alike and both instruments are equally near their respective ears, the voice apparently proceeds from in front of the observer.

